# Wearable Stretch Sensors for Human Movement Monitoring and Fall Detection in Ergonomics

**DOI:** 10.3390/ijerph17103554

**Published:** 2020-05-19

**Authors:** Harish Chander, Reuben F. Burch, Purva Talegaonkar, David Saucier, Tony Luczak, John E. Ball, Alana Turner, Sachini N. K. Kodithuwakku Arachchige, Will Carroll, Brian K. Smith, Adam Knight, Raj K. Prabhu

**Affiliations:** 1 Neuromechanics Laboratory, Department of Kinesiology, Mississippi State University, Mississippi State, MS 39762, USA; ajt188@msstate.edu (A.T.); snk128@msstate.edu (S.N.K.K.A.); aknight@colled.msstate.edu (A.K.); 2 Department of Human Factors & Athlete Engineering, Center for Advanced Vehicular Systems (CAVS), Mississippi State University, Mississippi State, MS 39762, USA; burch@ise.msstate.edu; 3 Department of Industrial & Systems Engineering, Mississippi State University, Mississippi State, MS 39762, USA; ppt25@msstate.edu (P.T.); smith@ise.msstate.edu (B.K.S.); 4 Department of Electrical & Computer Engineering, Mississippi State University, Mississippi State, MS 39762, USA; dns105@msstate.edu (D.S.); jeball@ece.msstate.edu (J.E.B.); woc17@msstate.edu (W.C.); 5 National Strategic Planning and Analysis Research Center (NSPARC), Mississippi State University, Mississippi State, MS 39762, USA; tluczak@nsparc.msstate.edu; 6 Department of Agricultural and Biomedical Engineering, Mississippi State University, Mississippi State, MS 39762, USA; rprabhu@abe.msstate.edu

**Keywords:** wearable devices, motion analysis, fall prevention, human factors, occupational falls

## Abstract

Wearable sensors are beneficial for continuous health monitoring, movement analysis, rehabilitation, evaluation of human performance, and for fall detection. Wearable stretch sensors are increasingly being used for human movement monitoring. Additionally, falls are one of the leading causes of both fatal and nonfatal injuries in the workplace. The use of wearable technology in the workplace could be a successful solution for human movement monitoring and fall detection, especially for high fall-risk occupations. This paper provides an in-depth review of different wearable stretch sensors and summarizes the need for wearable technology in the field of ergonomics and the current wearable devices used for fall detection. Additionally, the paper proposes the use of soft-robotic-stretch (SRS) sensors for human movement monitoring and fall detection. This paper also recapitulates the findings of a series of five published manuscripts from ongoing research that are published as Parts I to V of “Closing the Wearable Gap” journal articles that discuss the design and development of a foot and ankle wearable device using SRS sensors that can be used for fall detection. The use of SRS sensors in fall detection, its current limitations, and challenges for adoption in human factors and ergonomics are also discussed.

## 1. Introduction

Wearables are often defined as “technologies used to measure various physiological and kinematic parameters by being sported or borne by the user” [1,2]. The purpose of wearable technology or devices is to assess human performance—that is, biomechanical or physiological in nature—or for monitoring specific events of human movement in daily living, athletic, clinical, or occupational populations. The advantage of wearable devices is that they allow for monitoring human performance continuously and in environments that are outside of a laboratory or clinic with ease. This advantage can help to assess, diagnose, treat, and prevent injuries, especially in occupational settings where there is an elevated risk for work-related injuries. According to the National Safety Council (NCS), in 2017, a total of 4.5 million work-related medically consulted injuries and 4414 preventable work-related deaths occurred in the United States [3]. Falls are the leading cause of both fatal and nonfatal injuries in occupational populations [4]. Falls and fall-related injuries can be attributed to postural instability caused by an induced loss of balance and failure to recover from the imbalance, which commonly occurs in hazardous occupations. The Bureau of Labor Statistics (BLS) reported that, in 2017, from a total of 5147 fatalities, 887 were attributed to falls, slips, and trips, and a total of 227,760 cases of nonfatal workplace injuries were due to falls (47,180 falls to a lower level, 142,770 same-level falls, and 33,720 slips/trips), with a high incidence rate especially in construction (24,160 falls) and manufacturing (22,010 falls) [4]. Moreover, the innate dangers in hazardous occupations such as construction, manufacturing, transportation, warehousing, mining, quarrying, and healthcare services, as well as emergency responders—such as firefighters, law enforcement, and military—predisposes greater risks for occupational injuries [5,6,7,8,9,10]. In addition to the hazardous work conditions, physical exertion mandated by the occupational tasks creates greater demands on the human postural control system, thereby increasing the risks of falls [11]. Furthermore, the economic and financial costs associated with work-related accidents and injuries pose a significant threat and burden to the nation and the world. In 2017, the NCS reported $161.5 billion as an estimated cost for work-related injuries in the United States [3]. The constant increase in injury, illness, and accident rates in the workplace warrants the successful implementation of safety practices that are evidence-based. This further warrants the need for new innovating and emerging research to minimize workplace fall-related accidents.

With greater advancements in technologies, there are multiple tools and equipment, such as camera-based systems, ambient sensors, and various types of wearable sensors, that are helpful to detect falls and near falls in an attempt to reduce fall-related injuries [12]. In this context, monitoring employees through wearable sensors for potential falls or near-falls during occupational activities will aid not only in detecting falls but can also help in pre-fall and post-fall interventions [13]. The traditional fall prevention technologies such as the camera-based systems, ambient systems, and fall alert sensor systems identify falls after they have occurred and help to contact emergency services. Whereas, wearable technologies are used as fall monitoring and detection systems that help to identify discrete fall or near-fall events over the course of the day [13] and can be extremely beneficial, especially during high fall-risk occupational tasks. With the ever-increasing fall risk in hazardous occupations, there is a need to mitigate such injuries and improve safety.

Although there are multiple sensors being used for human monitoring, the advent of wearable stretch/strain sensors (WSS) that are either worn or attached to the skin is more recent. Hence, this paper provides an in-depth review of the current WSS technology for human movement monitoring by addressing their uses, applications, findings, limitations, and future scope. This paper also recapitulates the findings of a series of five published manuscripts from ongoing research that are published as Parts I to V of “Closing the Wearable Gap” journal articles [1,14,15,16,17] that discuss the design and development of a foot and ankle wearable device using wearable soft robotic stretch (SRS) sensors that can be used for fall detection. The use of SRS sensors in fall detection, its current limitations, and challenges for adoption in human factors and ergonomics are also discussed.

## 2. Wearable Technology

The wearable technologies used to capture metrics about human performance often receive most of the focus. Performance assessment wearables are largely responsible for the booming growth in wearable user consumption, which began at the 2014 Consumer Electronics Show and is expected to hit $34 billion [18] to $40 billion [19], with an estimated 485 million devices shipped, in 2019. The purpose of most wearables across many environments is to paint a complete picture of what continuous work outside of the lab does to the human “athlete”—be they sports athletes, industrial athletes, tactical athletes (war fighters and first responders), or even the at-risk athletes who are in rehabilitation or longer-term treatment. The mass popularization of smartphone and mobile device technology has enabled the miniaturization of data-capturing sensors and other processing and storage components, such that computers can be embedded into clothing and other noninvasive locations on a person while they are actively performing a task. How individuals work and, in turn, how that work affects them can be more effectively optimized, quantified, and tracked. More advanced wearable electronic sensors exist that range in their applications from detecting biomechanical movements [14,16], haptic and touch perception [20,21,22], human physiological responses [23], and even bioinspired sensors that mimic the functions of the human sensory nervous system [24]. These advanced wearable electronic sensors were developed and validated predominantly for bridging the gap in the human-machine/computer interface literature and their applications [20,22]. These sensors aid in capturing precise human responses and aid multiple aspects of applications ranging from clinical, rehabilitation, athletic, and occupational populations.

## 3. Wearable Stretch Sensors

The WSS have numerous applications that involve motion capture studies. For body strain measurements, these can be integrated onto clothing or directly laminated on human skin. Measurements ranging from minute skin motions induced by respiration and heartbeat to more significant human body strains like the bending or straightening of body joints can be obtained [25,26]. The information obtained from these sensors can be used to evaluate body movements, posture, and performance of the player during sports activities [26,27]. The information recorded could be useful for monitoring the body performance and wellness analysis of an individual. Another application of SRS involves mounting them on the knee joint [25,28,29]. This helps in gaining information about different knee patterns, such as walking, running, jumping, squatting, and various other activities. WSS are beneficial for continuous health monitoring, rehabilitation, and the evaluation of human performance.

### 3.1. Review of Wearable Stretch Sensors for Human Movement Monitoring

A brief review of a variety of such WSS and skin-mounted sensors with their broad applications in human motion detection have been summarized in Table 1. The review table surveys various studies conducted on the application of WSS that include both resistive and capacitive types of sensors. The table highlights the potential application of these wearable sensors as motion-capturing devices and comprises of a review of human movement monitoring, gait analysis, and other movement-based applications using such WSS. Table 1 also includes information about the current challenges and limitations in the use of skin mountable and wearable sensors for body-integrated applications [30,31,32,33,34,35,36,37,38,39,40,41,42,43].

### 3.2. Design and Development of Wearable Devices Using Soft Robotic Stretch (SRS) Sensors for Human Movement Monitoring

**Figure 1:**
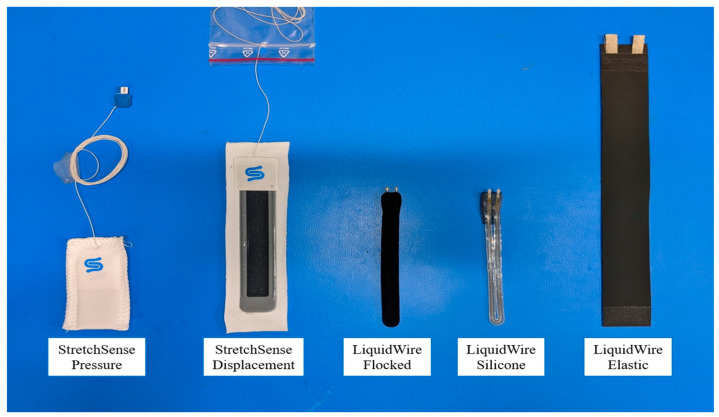
Different wearable stretch sensors used by the current research team. From left to right: (1) StretchSense Pressure Sensor, (2) StretchSense Displacement Sensor, (3) LiquidWire Flocked Sensor, (4) LiquidWire Silicone Sensor, and (5) LiquidWire Elastic Sensor.

**Figure 2:**
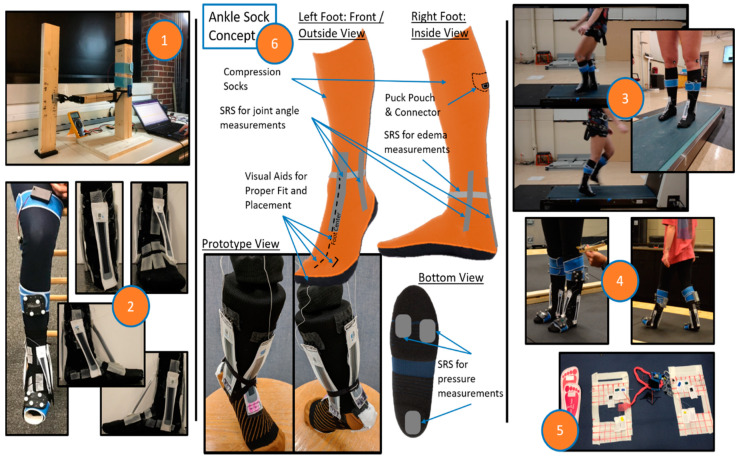
A pictorial representation of past and future works from the “Closing the Wearable Gap” (CWG) journal article series (Parts I to V). (1) CWG Part I—Mobile Systems for Kinematic Signal Monitoring of the Foot and Ankle, (2) CWG Part II—Sensor Orientation and Placement for Foot and Ankle Joint Kinematic Measurements, (3) CWG Part III—Use of Stretch Sensors in Detecting Ankle Joint Kinematics during Unexpected & Expected, Slip & Trip Perturbations, (4) CWG Part IV—3D Motion Capture Cameras Versus Soft Robotic Sensors Comparison of Gait Movement Assessment, (5) CWG Part V—Development of Pressure-Sensitive Sock Utilizing Soft Sensors, and (6) CWG—future iteration of an ankle sock concept to monitor human movement.

While several wearable devices that incorporate different types of sensor technology exist for fall detection, they have their own limitations, such as inertial measurement unit (IMU) distortion, reliability, and high financial costs [44,45]. Moreover, there is a constant need for the design and development of novel wearable technology to combat the increasing threat of falls and fall-related injuries in occupational settings. Our research team was tasked with the design and development of a wearable device using soft-robotic stretch (SRS) sensors capable of capturing the human joint movement kinematics, specifically at the ankle joint in the lower extremity. The research team has since then published a series of five papers under the “Closing the Wearable Gap” series: Part I to Part V [1,14,15,16,17], which discuss the design, development, and testing of the foot and ankle wearable device. Specifically, the Parts I and II papers tested the reliability and feasibility of using SRS on both a mechanical ankle joint device and on human participants [1,14]. The SRS are thin strap-like electronic sensors that produce a linear change in voltage recorded either in resistance (LiquidWire, Beaverton, Oregon, USA) or capacitance (StretchSense, Auckland, NZ, USA) when they are stretched (Figure 1). Subsequently, when the SRS were fixed on the anterior, posterior, medial, and lateral sides of the foot and ankle segments spanning across the ankle joint axis, they stretch during all four degrees of freedom of the ankle joint, plantar flexion, dorsiflexion, eversion, and inversion movements, respectively. The change in voltage was correlated to the change in the ankle joint range of motion angles using traditional electric goniometers as well as using the gold standard 3D motion capture system. The results from these papers identified significant linear models and validated with significant goodness-of-fit when compared to the gold standard 3D motion capture system [1,14]. The linearity of the stretch from the SRS was reported to have an R^2^ value of 0.99 in the Part I paper and an R^2^ value of 0.95–0.99 in the Part II paper. Thus, the Parts I and II papers (Figure 2) demonstrated that the SRS sensors could be used as a potential wearable device to detect ankle joint kinematics in both sagittal and frontal movements of plantar flexion/dorsiflexion and inversion/eversion movements, respectively. However, the movements in these two studies were performed one at a time from a static, non-weight-bearing condition. The need for assessing the use of SRS sensors in dynamic movements, especially fall detection, was necessary. The critical advancement of studies exploring more complex movements lead to the Parts III, IV, and V papers (Figure 2) [15,16,17], the next projects investigated by the research team, which are explained further with in-context of WSS in fall detection, their applications, limitations, and future scope.

Although different types of sensors are being used for fall monitoring and detection, the placement of these sensors on the human body have been limited predominantly to the torso and lower extremities [46], and body-worn sensors used for fall detection have also been traditionally placed on the waist/hip or as trunk attachments [46]. Occasionally, wearable sensors such as accelerometers that are placed on the head and neck have also been utilized that detect the acceleration changes of the head in the event of falls [47]. However, based on the postural stability model suggested by Winter (1995), the human body is considered as an inverted pendulum, with the axis of rotation pivoted at the ankle joint [48]. Subsequently, placing the SRS sensors across the ankle joint axis allows the researchers to monitor the kinematics of the ankle joint complex from an inverted pendulum model aspect. The SRS sensors placed on the anterior aspect of the feet stretches during plantar flexion, while the one placed on the posterior aspect of the feet stretches during dorsiflexion. Similarly, the SRS sensor on the lateral aspect of the ankle stretches during inversion, and the one on the medial aspect stretches during eversion. Correlating the linear change in voltage due to the stretch of the SRS with changes in the ankle joint range of motion in degrees quantified using 3D motion capture enables constant monitoring of ankle joint kinematics during human physical activity.

## 4. Wearable Sensors and Fall Prevention

### 4.1. Current Wearable Technology in Fall Monitoring and Detection

While camera-based and ambient systems have been used for fall detection based on changes in body movement and posture, movement inactivity detection, and head motion analysis, these solutions have their limitations, such as the obstruction of capture volume, privacy, false alarms, and battery life [49,50]. Wearable devices have been successfully implemented and used to assess human physical activity in multiple populations [12]. More specifically, wearable or body-worn sensors have become the preferred choice of technology for fall monitoring and detection [12,46,51] due to their high precision, less time commitment, easy access, feasibility, and administration [50]. These wearable devices include inertial measurement units (IMUs), accelerometers, gyroscopes, magnetometers, pedometers, electric goniometers, and foot pressure sensors [12,49,50,52,53,54]. More often than not, these physical, wearable sensors have been used along with smartphones and applications to provide an effective wearable fall-detection device and system [55,56,57,58,59].

### 4.2. Use of SRS Sensors for Fall Detection

With the SRS sensor design completed, the Parts III and IV papers address the validation of the SRS sensors during dynamic tasks specific to slip and trip perturbations [15] and while walking on sloped surfaces [16] (Figure 2). In Part III of the series, participants wore SRS sensors and were subjected to both unexpected and expected postural perturbations imparted by the sudden starting and stopping of a treadmill belt from the static nonmoving position. All sensor data were compared to ankle joint plantar flexion/dorsiflexion quantified using a 3D motion capture system. Trials during which the treadmill belt moved forward in relation to the individual were used to create slip perturbations, and the trials during which the treadmill belt moved backward in relation to the individual were used to create trip perturbations. The unexpected trials were created when the participants were not informed of the upcoming perturbation type and time and were provided randomly within 30 s of static stance. The expected trials were when participants were informed of the upcoming perturbation type and time and were counted down numerically to provide the perturbation. The use of both slip and trip perturbations and both unexpected and expected perturbations were in an attempt to assess the validation of the SRS sensors during different types of fall detection and for the validation of the behavior of the SRS sensors during rapid unexpected and braced expected falls. Adjusted R^2^ and root mean square error (RMSE) were used to validate the SRS sensor data with the 3D motion capture ankle angle kinematics. The results from the study identified a medium-to-high adjusted R^2^ value (R^2^ = 0.60) and a low RMSE value (<4 degrees), thus suggesting a moderate-to-high accuracy with minimal errors in comparing the SRS sensors against the 3D motion capture system during these different postural perturbations. For verification, R^2^ and RMSE have shown to be valuable assessment methods for kinematic and kinetic data related to sports and dynamic movements [60]. Thus, the findings suggest that the SRS sensors could be a feasible option in detecting ankle joint kinematics during slip and trip-induced falls [15].

As a follow-up to the slip-trip testing, the Part IV paper addressed the validity of SRS sensors during walking both on a flat surface and a tilted surface [16]. All four SRS sensors for capturing plantar flexion/dorsiflexion and inversion/eversion were used to measure ankle joint kinematics simultaneously. Participants walked with a self-regulated pace on a custom-built wooden platform, and a total of 12 gait trials, with six on each surface (flat and titled), were collected to acquire a total of 24 gait cycles for each participant. In addition to the previously used adjusted R^2^ and RMSE, the mean absolute error (MAE) was calculated to validate the SRS sensor data with the 3D motion capture ankle angle kinematics for all four degrees of freedom. The findings indicated that all four SRS sensors provided a successful fit identified by a high adjusted R^2^ value (R^2^ = 0.854) and lower MAE (MAE = 1.54) and RMSE values (RMSE = 1.96), suggesting that SRS sensors could be a feasible option to capture ankle joint kinematics both on flat, as well on tilted, surfaces [16]. The validity of the SRS sensors during dynamic walking on tilted surfaces from Part IV [16] and during rapid slip-trip perturbations from Part III [15] suggest that SRS sensors could be a new wearable device that can detect ankle joint kinematics in fall-prone conditions, where the human body is subjected to different perturbations that destabilize the body postural stability (Figure 2).

Finally, a comprehensive fall detection system could not just rely on capturing joint kinematics, and capturing kinetics, especially forces from the feet during ground contact, need to be prioritized as well. Subsequently, Part V of the paper series [17] successfully attempted to develop a pressure-sensitive sock using a compressible variation of the same SRS sensors (five sensors) placed on the sole of each foot enclosed in a sock. Pressure (kPa) from the soles of the feet were quantified using pressure cells (BodiTrak™ Vista Medical, Winnipeg, MB, Canada), as well as with ground reaction forces (N) from dual-force platforms (Kistler™ Novi, MI, USA), compared to the compressible SRS sensors during different activities such as squatting, shifting weight from left to right, and shifting weight from heels to toes. Correlations—mean R^2^ and mean RMSE—were used to compare the changes in pressure of compressible SRSs, changes in pressure on the BodiTrak™ Vector Plate, and changes in force on the Kistler™ Force Plates. The results identified a positive linear relationship between the compressible SRS sensors and BodiTrak™, while the comparison to the force plates was inconclusive [17]. Based on the findings, the compressible SRS sensors were still identified as an effective option to capture the pressure distribution from the sole of the foot during ground contact, which serves a vital purpose in identifying the weight-bearing status of the specific lower extremity in the event of falls or near-falls. In the context of a fall detection system using the SRS sensors, the need for kinetic data from the feet during ground contact, in addition to the ankle joint kinematics, is further explained under the current limitations sections.

## 5. Limitations and Future Scope

### 5.1. Limitations to Wearable Stretch Sensors

There are several limitations and future developments to be considered when working with SRS. Factors such as placement of the sensors, perception of space, body diversity in anthropometry and movements, attachments, containments, sensory interactions, aesthetics, and long-term use play very vital roles on the wearability of the sensors. The wearable SRS must be durable enough to perform consistently for the time and conditions of use in which they are expected to collect data. Some of the challenges faced when using SRS include sliding and distortion of the sensors during skin deformations, impact with an object and stress-induced movements, leading to underestimation of the actual motion [32,34,35,37]. Exploiting textile engineering techniques, collaboration between designers and engineers would help to improve smart clothing designs from a noninvasive and comfort perspective. Manufacturing clothes with close-fitting garments would help to minimize sensor movements and drift, thereby improving accuracy.

Another possible source of error for an SRS-based device was nonlinearity of the wearable sensors under compressive force. Hysteresis, which can be defined as a natural reluctance for the sensors to return to the original length after removal of a load, and the nonlinearity of sensors due to such changes in its material properties further add to the complexity and difficulties with sensor errors [61]. The error concerning hysteresis and nonlinearity was observed as a major drawback for all resistive-type wearable sensors. Efforts have been made to add a pressure-sensing element on top of the strain-sensing element, allowing the sensor to detect compression in addition to the strain [62]. Under dynamic loading conditions, hysteresis was observed in the sensor response, which can be due to the ways sensors were integrated to the body or might have been caused due to the viscoelastic nature of the polymers. Carefully selecting the location of the sensors—that is, moving their location from directly on top of the joints to more soft and flat areas of the body—would help reduce the pressure effect as well; however, the changes concerning relocation of the sensors would make the design process more complicated. Recently, deep-learning methods have been proposed for full-body motion sensing to solve the problems of nonlinearity and hysteresis [35].

The accuracy of the sensor readings highly depends on the care taken during the calibrations. Even a slight difference in the calibration leads to drastic changes in the joint angle prediction [30,32,36]. There were differences noted in the actual and predicted angles. Various approaches have been proposed using different kinds of devices, such as an electro-goniometer, IMUs, and camera-based optical systems. However, these solutions all still had some limitations, such as inaccuracy in detecting multiple degrees of freedom for joint motions, errors with high-speed motions, and space confines [34,35,37]. Additionally, discomfort of the wearers due to the attachment of rigid electronics on garments or skin were reported. Based on studies concerning calibration datasets during motion sensing, a robust calibration process for motion capture using computational methods involving machine-learning and deep neural network systems is required to deal with the issues more effectively [35,63].

### 5.2. Current Limitations of the Stretchable SRS and Measures to Minimize Errors in Fall Detection

Even though the SRS sensors were validated against a motion capture system and identified as a potential fall detection sensor, both during unexpected and expected slip and trip perturbations [15], as well as during walking on sloped surfaces [16], a few limitations still exist. The data from just the four SRS sensors on the foot and ankle segment used to identify ankle joint kinematics [15,16] would not essentially be a comprehensive fall detection system. Deviations from acceptable changes in ankle joint kinematics are used to detect any aberrant movements during the course of a physical task. For example, during a slip-induced fall, a change of 30 degrees in plantar flexion could be evident. However, while going up on one’s toes during a reaching to a height task, a similar change of 30 degrees in plantar flexion is possible. Hence, to differentiate a fall-induced angular displacement from a task-induced one, the rate of change of angular displacement, angular velocity, should also be quantified, as slip or trip-induced falls tend to have a faster angular velocity. Yet, assessing kinematics of the ankle joint alone may still not be sufficient to have a precise fall detection system. The addition of SRS sensors repurposed as a pressure-sensitive sock to detect pressure underneath the sole of the foot will aid in the identification of the weight-bearing status of the lower extremities [17]. Subsequently, the knowledge of the pressure distribution during weight-bearing activities and the absence of pressure during non-weight-bearing conditions can aid in identifying the context in which the ankle joint is moving. Similarly, during weight-bearing activities such as walking, the presence of different ratios of pressure distribution can aid in detecting the different subphases of the stance phase of the gait cycle. As such, the presence of pressure distribution in one foot but the absence in the other with periodic repetitions can indicate the stance phase and swing phases of the gait cycle during walking. However, the absence of pressure distribution in both feet accompanied by extremes of the joint range of motion kinematics can potentially indicate a fall event. The current limitations of the SRS sensors can be minimized with the above-discussed measures, as well as with the addition of different types of sensors, as discussed in the below section on future SRS sensor development. However, repeated testing both in the laboratory and in the field, especially in hazardous occupational environments, such as in the roofing and construction industry, is essential.

### 5.3. Future Stretchable SRS Development for Fall Detection

The future of SRS sensors being incorporated into fall detection devices, especially in addition with other types of sensors working in unison, can provide high-quality data of human movement and the utmost precision in detecting and preventing falls and alarms in fall detection. The existing, wearable system of four SRS sensors on the ankle joint axis to measure the ankle joint kinematics and five sensors on the sole of the foot to measure foot pressure can still be enhanced by adding other sensors to make a comprehensive, wearable fall detection system. For example, IMUs and accelerometers have been previously used to detect abnormal movement patterns of the body [49,64] and electromyography (EMG) recordings, especially from the lower extremity muscles, to detect pre-falls to the ground in the forward, backward, and lateral directions [65]. While these wearable devices have been used in isolation, the impact of a comprehensive, wearable fall detection system utilizing different sensors is still lacking. Finally, the use of SRS sensors allows them to be sewn into compression garments as they contour around the shape of the body, potentially paving the way for future smart garments of fall detection in the workplace.

## 6. Challenges to the Use of Wearable Devices in the Workplace

Wearable technologies are being increasingly promoted and used in the workplace for employee safety and injury prevention [66]. Specific to fall prevention in the workplace, even though personnel protective equipment (PPE) such as fall-harnesses and fall-arrest systems are mandated for fall-risk workplaces, the use of wearable technology provides an opportunity to continuously monitor the safety status of the employees and to find at-risk employees who are more prone to fall. Individuals who might have their postural control system compromised due to any neurological or musculoskeletal disorders, due to the hazardous working conditions (such as working in fall-risk conditions, working in awkward postures, and improper or poor PPE availability and use) or due to the inherent hazards of the occupation (such as physical and mental fatigue, overexertion, etc.) can be identified early before events of falls and given appropriate training and safety precautions. While the use of wearable technology seems to aid the well-being of the employee and minimize the financial cost to the organizations due to fall accidents, there are still challenges to their adoption. A recent study by Schall et al. (2018) identified barriers such as employee privacy, compliance, wearable device’s durability, and the cost-benefit ratio, which have prevented the widespread adoption of wearable technology in the workplace [66]. Even though the study by Schall et al. (2018) did not focus on specific types of wearable devices pertaining to fall detection, the perception of the identified barriers and, in turn, the adoption remains a challenge in the workplace. Subsequently, the incorporation of multiple types of sensors specific to the occupational tasks can provide a comprehensive employee monitoring system to prevent injuries and promote safety. Reducing the injury risk and increasing employee satisfaction, wellness, and productivity have been identified as potential benefits of using wearable devices in the workplace [66]. Meanwhile, organizations that intend to adopt wearable technology need to focus on workplace safety and inform and support the employees of wearable technology and address the barriers for adoption [66,67]. The implementation of SRS sensors for fall monitoring and detection will also face the same barriers as other wearable technologies. Multiple field-testing and awareness creations of the scientific community will aid in breaking the barriers for adoption and increase the use of wearable SRS fall detection systems [66,67].

## 7. Conclusions

This paper provides a review of the current WSS, a summary of the current research team’s efforts to design, develop, and test a foot and ankle wearable device with the use of a novel SRS sensor and, subsequently, propose this wearable device as a potential fall detection system in the field of human factors and ergonomics, while addressing the limitations, future scope, and challenges of such wearable devices in the workplace. The SRS sensor has been validated in five different studies published as a series of Parts I to V papers in the “Closing the Wearable Gap” research. Over the course of the design and development of this wearable device, foot and ankle joint kinematics and kinetics captured by the SRS sensors were validated against an electronic goniometer, 3D motion capture systems, pressure mats, and force platforms. Specific to fall detection, the foot and ankle wearable device using the SRS sensors was identified as a promising technology to detect falls by assessing ankle joint kinematics during unexpected and expected slips, trips, and walking on tilted walkways. Thus, based on the current available literature, their findings, limitations, and future scope, this paper attempts to “Close the Wearable Gap” on WSS and their use in human movement monitoring and fall detection.

## Figures and Tables

**Table 1:** A review of different studies assessing human movement with the use of wearable stretch sensors with descriptions of the study applications, tests conducted, and findings, as well as limitations and future scope.

Study	Uses/Application	Tests Conducted	Findings	Limitations	Future Scope
**(Kramer et al., 2011)** [30]	Position measurement, motion detection, joint rotation incorporated in gloves to monitor hand motions	Curvature sensing, stretch sensing, and combination of curvature and stress sensing. Finger positioned between 0° and 90° and the change in resistance was recorded. Two tests conducted: (1) the finger bends at 90° and stays there for a while: (2) rapid bending and straightening. No. of participants: n/a	Elastomer based curvature sensors allow mechanically non-invasive measurements of human body motions and kinematics. Owing to high stretchability, the sensors conform to the host bending without interfering with the natural mechanics of motion.	Accuracy of the sensor highly dependent on calibration, slight difference in calibration leads to drastic change. Changes in viscoelasticity of the sensor recorded	Integration of hyper elastic pressure and curvature sensors with integrated circuit. Shape mapping elastomer sheets.
**(Huang et al., 2017)** [32]	Used in wrist rehabilitation, to increase the competitiveness of some sports by capturing and analyzing the joint motions, to obtain better effectiveness and accurate feedback of wrist motion for training in sports.	Five Degree of Freedom tested: flexion, extension, pronation, supination, ulnar deviation. No. of participants: 4	A comfortable, portable and accurate wrist motion capture system. Decoupling algorithm proposed to solve the coupling problem of the measurements Used for analysis of athletic training performance, rehabilitation training, virtual reality system and control manipulators of robotic systems.	Sliding of the sensors during skin deformation led to underestimation of the actual motion. Positioning repeatability is low. For the decoupling algorithm, if the Dielectric Elastomers (DES) system is worn by different people, the co-efficient matrix would change, and the system needs to be recalibrated. 2D measurement used in order to evaluate decoupling.	Focus on improving the measurement precision and making the system more comfortable to wear. Integration of sensors into tight fitting clothing can help eliminate positioning errors of the sensor building of 3D motion evaluation system to validate decoupling algorithm. Optimizing the dimensions to of DES to maximize sensitivity.
**(Al-Nasri et al., 2019)** [33]	Studying the neck range motion for various neck related ailments.	Participants fitted with the commercial stretch sensitive C-Stretch tape along the sternocleidomastoid muscle (SCM) on both sides of the neck with the wire-end of the tape closer to the clavicle. Participants were asked to actively rotate their heads to the left from neutral and then right for a total of five left-to-right rotations. No. of participants: 2	Assessment of commercially available capacitive stretch sensitive sensors for real time monitoring of cervical range of motion. Usability of wearable sensors as a safe alternative to assess neck range of motion for clinical applications.	Anthropometric variables do not allow optimal use of sensor position and type for capturing joint motion. The length of sensor does not exceed to the full length of the sternocleidomastoid muscle for most people	Safe alternative to assess real world neck range of motion for clinical application. Protocol for adhesion with a larger sample size to reduce the accuracy error below the acceptable threshold. Implementation of a more accurate method for fitting curvilinear nature of movements involved in rotations.
**(Shen et al., 2016)** [31]	Clinical use for finger kinematic analysis and hand function evaluation.	Sensing materials’ ability to bend and stretch are tested. Repeatability is tested. No. of participants: n/a	An analytical study to design the sensor with bending and stretching features enabling the sensor to be implemented in measuring human motions where a large amount of skin stretch is involved. Two sensor gloves designed and fabricated based on the proposed soft bending sensor for different applications.	Hysteresis and visible fluctuations observed, caused by sensor elongation and unstable connection.	Improve repeatability by reducing hysteresis and introducing new algorithm. Using the same system to measure bending angles of wrist and elbow.
**(Totaro et al., 2017)** [34]	Used to address joint motion detection in and off sagittal plane. The capacitive elements are sensitive to pressure solicitations allowing discrimination between strain and pressure. In rehabilitation field, can be used to provide feedback about abnormal postures. Can be used to monitor and improve athlete’s performance and track user movement in gaming.	For knees: Standing, sitting, squatting, running, walking. For ankle: dorsi/plantar flexion, adduction/abduction, complete foot rotation. No. of participants: n/a	Smart garments developed for lower limb motion detection embedded with readout electronics for retrieving movement of specific joint. Smart anklet designed to address joint motion detection in and off sagittal plane. Garments show high accuracy in movement detection with root mean square error less than 4° in worst case situation.	Sliding of braces after activities. Washability of the modules. Partial detachment of sensors due to the stress induced by the movements	Development of customized smartphone application, which helps guiding the user on different positions of foot needed for calibration, acquire sensor outputs and implement polynomial fitting for measuring full ranges. Using five sensors at the knee to detect internal/external rotation, and its abduction/adduction angles. Sewing of fabric conductive electrodes into the garment to isolate it from the user and outside world. Using knee & ankle modules together to provide biomechanical information about lower limb movement.
**(Kim et al., 2019)** [35]	Use of deep learning for full body motion sensing, biomechanics study and rehabilitation. Use of Deep Full Body Motion Network (DFM-Net)	Three types of activity data set: squats, bend and reach and windmill motion, four sets of each. Use of deep neural networks. No. of participants: n/a	Use of deep learning for full body motion sensing, significantly increasing efficiency in calibration of the soft sensor and estimation of body motions. Deep learning-based calibration and mapping method shows a higher accuracy than traditional methods based on mathematical estimations.	Nonlinearity in response and hysteric loops during flexion and extension of knee joint. Alignment, anchorage and deformation of human body resulting in difference in magnitude and pattern as well as noise in the output signals from shoulder joints.	Development of an improved calibration model that can reuse pre-trained model parameters to simplify calibration procedures.
**(Yi et al., 2015)** [40]	A stretchable-rubber-based (SR-based) triboelectric nanogenerator (TENG) device, integrated into a sensor system, capable of detecting movements in different directions. The SR-based TENG attached to the body to detect breathing and joint motion	The SR-based TENG exploits the shape/length expansion of the rubber, which induces an in-plane charge separation and results in a potential output current produced by periodically stretching and releasing the rubber. This unique working principle of TENG was confirmed by numerical calculations and controlled experiments. No. of participants: n/a	Based on its motion sensing capability and high elasticity of the rubber, the SR-based TENG can be mounted onto a human body; and a self-powered health monitoring system could be realized for detections of physiological activities and joint movements. The SR based TENG can distinguish the bending angle of the knee, and accurate bending rate of the knee can also be acquired.	The theoretical vertical gap between the rubber and aluminum affects the charge transfer process.	New design opportunities for TENG with great potential for applications in robotics, entertainment, sports, medical diagnosis, medical treatment. Act as functional sensor to detect various kinds of signals.
**(Liao et al., 2019)** [43]	Strain sensors with both high stretchability and high sensitivity, fabricated based on cluster-type microstructures (CM) by using nozzle jet printing method.	Test were conducted by applying 00% and 160% stretching strains, to confirm the robustness of CM sensors. CM sensors were attached to multiple limb joints to monitor their activity. Knee bending detection was performed by the CM sensors, including bending, half bending and straight states. No. of participants: n/a	The CM strain sensor possesses a high gauge factor up to 2700 and a wide sensing range of 160% strain. Rapid response time is 18ms and response stability > 10,000 strain cycles were conductive to CM strain sensor to perform well in both static and dynamic conditions. The sensor yields a significant information about joint movement.	The materials and devices fabrication are a tedious process. The materials are required to be pre-stretched before nozzle jet print machine deposits silver ink.	Can be envisioned and expanded further to the exploitation of wearable electronics. Developing fiber-based sensors to achieve higher performances in future.
**(Chen et al., 2016)** [39]	Strain sensors with ultra-high sensitivity under microstrain having numerous potential applications in heartbeat monitoring, pulsebeat detection, sound signal acquisition and recognition.	Novel hybrid particles through coprecipitating silver nanowires (AgNWs) and graphene oxides (GO) were fabricated and two-part strain sensor was developed after a simple reduction, vacuum filtration and casting process. Cycle testing was taken for strain sensors under three different strains to investigate its practical application for sensing. No. of participants: n/a	The strain sensors show good response to bending, high strain resolution and high working stability and successfully used in detection of microstrain such as daily physical vibrations, wrist pulses, and sound signal recognition. Sensing mechanism under strain results in high gauge factor of the strain sensor. The strain sensors have an accurate response on various strains and good mechanical stability.	Any difference in AgNWs and GO proportions can lead to errors in coprecipitation.	Applications in sensing bending deformations. Can be used in detection of physiological signals and health monitoring.
**(Liao et al., 2018)** [41]	Intelligent glove assembly using textile strain sensors, capable of detecting and translating full range of fingers’ bending into wireless control commands.	A wearable intelligent glove assembled with multiple textile strain sensors, using facile stencil printing method. A deformation and fracturing mechanism investigated to check the effects of loading and unloading on the strain sensors. Microcontroller unit used for signal conditioning and Bluetooth transceiver used for signal communication. Successful demonstrations conducted to detect short bending and long bending of fingers and hand gestures, towards human machine interface applications. No of participants: n/a	The textile sensor possessed ultrahigh sensitivity with a relatively wide sensing range and gauge factor estimated to be approximately 2000. The textile strain sensors worked even when stretched up to 60% its original length. Simple signal processing unit with Bluetooth transceiver module allowing prompt transmission and translates fingers’ bending into wireless control commands. No additional signal conditioning required by the analog to digital converter circuit.	Stencil printing has a limited sensing range, which may restrict their application. Fingers need to work in sequence and cannot work simultaneously, as the targeted subject is unable to identify the control commands from intelligent glove when all fingers are working at the same time.	Higher sensitivity and larger detection range would be achieved by pre-stretching optimization processes of the textile. Development for future human-machine interface applications. Expanding the use of textile strain sensors in the field of Internet of Things.
**(Deng et al., 2019)** [42]	High stretchability and ultra-sensitivity of ultrasonic peeling vertical graphene/polydimethylsiloxane (UP-VGr/PDMS) sensors can be applied to various kinds of human physiological signal detectors. The sensors can detect sound amplitude as well as sound frequency, that is recognize the timbre of a sound.	Pull and compression tests, loading and unloading cycles to observe relative resistance changes. Analysis for human physiological signal detection—wrist bending, knee motions, different degrees of finger bending, pulse signal detection. Testing timbre recognition ability using cellphone as a sound source and applying continuous constant loudness soundwaves of varying frequencies. No. of participants: n/a	The UP-VGr/PDMS strain sensors have high stretchability, up to 100% and high sensitivity (Gauge Factor > 10,000 at 100% strain) the sensors have the ability of high timbre recognition without waveform distortion for frequencies as high as 2500Hz. Demonstrations presented to highlight the sensors’ potential as wearable device for human motion, pulse and sound timbre detection.	Hysteresis behavior of strain sensor observed under loading cycles with 1 mm s^−1^ loading and unloading speeds. Little attenuation observed under unloading process.	Owing to good response to small angular changes and relatively high sensitivity, the sensors can be used as a highly selective sound detector, such as speech pattern recognition system. Sensors with timbre recognition ability can be used for rehabilitation of hearing-impaired and speaking impaired people.
**(Liao et al., 2016)** [38]	Low cost stretchable, multifunctional sensor based on zinc oxide (ZnO) nanowires that can be stretched up to 150% and maintains the ability to detect strain, temperature and UV.	Field emission scanning electron microscopy used to study the sensor under strain up to 120%. Electrical properties of the stretchable sensors under dynamic and static strain were investigated. Temperature sensitivity of the multifunctional sensor tested at various strain conditions. Photoelectric property of stretchable UV sensors evaluated under various strain conditions. No. of participants: n/a	The electrical response of stretchable sensors remains unchanged through more than 10,000 cyclic loading tests at 3 Hz, possessing high stability and durability. The stretchable sensor shows high and stable signal-to-noise ratios. The stretchable temperature sensor under cyclic temperature tests between room temperature and 50 °C, is thermally stable without any strain. As stretchable UV sensors, the electrical signals respond and reset slowly.	Significant hysteresis behavior is observed associated with elastic loading. Maximum detection frequency is limited to 8 Hz which is contingent on the contact-separation speed of the small pieces of ZnO nanowires debris.	Obtain smaller cracks of ZnO nanowires debris to enhance the performance od the stretchable strain sensor. Potential application for temperature controlling devices and can be applied to a wide range of applications in human medical monitoring and sleep quality perception. Light weight and fiber shape of stretchable UV sensors paves a way to manufacture high stretchable and economical UV early warning devices and optical smart devices.
**(Lee et al., 2016)** [36]	Gesture recognition, motion monitoring, rotational angles of multi-axis joints.	Two stretch sensors attached along the skin affected by the rotation of the joint. Calibration process necessary to determine sensor axes to reduce misalignment to the axis of rotation. Shoulder flexion/extension and addiction/abduction were estimated. No of participants: n/a	A highly stretchable soft sensor which adheres to the skin directly to estimate multi axis joint rotation angles while providing comfortable physical interface.	Estimated shoulder movement show hysteresis behavior that result in error for fast movements, owing to viscoelastic property of sensor. Error in the calibration observed due to twisting effect of arm since sensor is directly attached to the skin (curved surface of the shoulder), inaccuracy caused by calibration method, non-linearity of the sensor.	The proposed system can be applied for motion monitoring system by direct attachment to skin without discomfort to the user.
**(Mengüç et al., 2014)** [37]	Study the hip, knee and ankle sagittal plane joint. Applications involve monitoring patient’s gait pathology for providing rehabilitation assistance, augment human performance by reducing the work required from biological muscles.	Tested in isolation for extreme extension to failure, moderate extension to 1500 cycles and extreme compression to failure. Three males under 30 tested for locomotion at 5 predefined speed over split belt treadmill. Kinematics collected using optical motion capture were synchronized to the data collected by the sensors, to validate the results obtained. No. of participants: 3	Systematic design and characterization of soft sensing suit for monitoring hip, knee and ankle sagittal plane joint angles. Developing soft sensing suit with careful consideration of interface between components such that the root means square error for walking at 0.89m/s was less than 5° and for running at 2.7m/s was less than 15°.	Sensitivity to surface pressure, mechanical hysteresis observed. Cross sensitivity to compression. Electrical path being cut due to microchannel collapse on application of pressure during compression. Joint angle measurement deviations observed with increase in locomotion speed.	Reduce material stiffness and mitigate compressor induced failure Using ionic liquids to improve biocompatibility as an alternative to liquid metal. Mechanically protecting sensors from redundant sensing to enable robust applications in field setting. Implication of a refined design with the use of discretized stiffness gradients to improve mechanical durability.

## References

[1] Luczak T., Saucier D., Burch V., Reuben F., Ball J.E., Chander H., Knight A., Wei P., Iftekhar T. (2018). Closing the Wearable Gap: Mobile Systems for Kinematic Signal Monitoring of the Foot and Ankle. Electronics.

[2] Luczak T., Burch R., Lewis E., Chander H., Ball J. (2019). State-of-the-art review of athletic wearable technology: What 113 strength and conditioning coaches and athletic trainers from the USA said about technology in sports. Int. J. Sports Sci. Coach..

[3] Injury Facts Work Safety Introduction. https://injuryfacts.nsc.org/work/work-overview/work-safety-introduction/.

[4] Injuries, Illnesses, and Fatalities. https://www.bls.gov/iif/.

[5] Chander H., Garner J.C., Wade C., Knight A.C. (2017). Postural Control in Workplace Safety: Role of Occupational Footwear and Workload. Safety.

[6] Chander H., Knight A.C., Garner J.C., Wade C., Carruth D., Wilson S.J., Gdovin J.R., Williams C.C. (2019). Impact of military type footwear and load carrying workload on postural stability. Ergonomics.

[7] Chander H., Knight A.C., Carruth D. (2019). Does Minimalist Footwear Design Aid in Postural Stability and Fall Prevention in Ergonomics?. Ergon. Des..

[8] Chander H., Knight A.C., Garner J.C., Wade C., Carruth D.W., DeBusk H., Hill C.M. (2018). Impact of military type footwear and workload on heel contact dynamics during slip events. Int. J. Ind. Ergon..

[9] Lyons K., Radburn C., Orr R., Pope R. (2017). A Profile of Injuries Sustained by Law Enforcement Officers: A Critical Review. Int. J. Environ. Res. Public. Health.

[10] Waldman H.S., Smith J.W., Lamberth J., Fountain B.J., McAllister M.J. (2019). A 28-Day Carbohydrate-Restricted Diet Improves Markers of Cardiometabolic Health and Performance in Professional Firefighters. J. Strength Cond. Res..

[11] Kincl L.D., Bhattacharya A., Succop P.A., Clark C.S. (2002). Postural Sway Measurements: A Potential Safety Monitoring Technique for Workers Wearing Personal Protective Equipment. Appl. Occup. Environ. Hyg..

[12] Mukhopadhyay S.C. (2015). Wearable Sensors for Human Activity Monitoring: A Review. IEEE Sens. J..

[13] Hamm J., Money A.G., Atwal A., Paraskevopoulos I. (2016). Fall prevention intervention technologies: A conceptual framework and survey of the state of the art. J. Biomed. Inform..

[14] Saucier D., Luczak T., Nguyen P., Davarzani S., Peranich P., Ball J.E., Burch R.F., Smith B.K., Chander H., Knight A. (2019). Closing the Wearable Gap—Part II: Sensor Orientation and Placement for Foot and Ankle Joint Kinematic Measurements. Sensors.

[15] Chander H., Stewart E., Saucier D., Nguyen P., Luczak T., Ball J.E., Knight A.C., Smith B.K., V R.F.B., Prabhu R.K. (2019). Closing the Wearable Gap—Part III: Use of Stretch Sensors in Detecting Ankle Joint Kinematics During Unexpected and Expected Slip and Trip Perturbations. Electronics.

[16] Saucier D., Davarzani S., Turner A., Luczak T., Nguyen P., Carroll W., Burch V., Reuben F., Ball J.E., Smith B.K. (2019). Closing the Wearable Gap—Part IV: 3D Motion Capture Cameras Versus Soft Robotic Sensors Comparison of Gait Movement Assessment. Electronics.

[17] Luczak T., Burch V., Reuben F., Smith B.K., Carruth D.W., Lamberth J., Chander H., Knight A., Ball J.E., Prabhu R.K. (2020). Closing the Wearable Gap—Part V: Development of a Pressure-Sensitive Sock Utilizing Soft Sensors. Sensors.

[18] Wearable Tech Market to be Worth $34 Billion By 2020. https://www.forbes.com/sites/paullamkin/2016/02/17/wearable-tech-market-to-be-worth-34-billion-by-2020/#6a1d4e013cb5.

[19] Reportlinker the Wearable Technology Ecosystem: 2016–2030—Opportunities, Challenges, Strategies, Industry Verticals and Forecasts. https://www.prnewswire.com/news-releases/the-wearable-technology-ecosystem-2016--2030--opportunities-challenges-strategies-industry-verticals-and-forecasts-300363863.html.

[20] Liao X., Wang W., Lin M., Li M., Wu H., Zheng Y. (2018). Hierarchically distributed microstructure design of haptic sensors for personalized fingertip mechanosensational manipulation. Mater. Horiz..

[21] Liao X., Song W., Zhang X., Zhan H., Liu Y., Wang Y., Zheng Y. (2019). Hetero-contact microstructure to program discerning tactile interactions for virtual reality. Nano Energy.

[22] Kim C.-C., Lee H.-H., Oh K.H., Sun J.-Y. (2016). Highly stretchable, transparent ionic touch panel. Science.

[23] Wang X., Gu Y., Xiong Z., Cui Z., Zhang T. (2014). Silk-Molded Flexible, Ultrasensitive, and Highly Stable Electronic Skin for Monitoring Human Physiological Signals. Adv. Mater..

[24] Liao X., Song W., Zhang X., Yan C., Li T., Ren H., Liu C., Wang Y., Zheng Y. (2020). A bioinspired analogous nerve towards artificial intelligence. Nat. Commun..

[25] Amjadi M., Kyung K.-U., Park I., Sitti M. (2016). Stretchable, Skin-Mountable, and Wearable Strain Sensors and Their Potential Applications: A Review. Adv. Funct. Mater..

[26] Coyle S., Morris D., Lau K.-T., Diamond D., Moyna N. Textile-Based Wearable Sensors for Assisting Sports Performance. Proceedings of the 2009 Sixth International Workshop on Wearable and Implantable Body Sensor Networks.

[27] Lam Po Tang S., Shishoo R. (2015). 8—Wearable sensors for sports performance. Textiles for Sportswear.

[28] Marsico M.D., Mecca A. (2019). A Survey on Gait Recognition via Wearable Sensors. ACM Comput. Surv..

[29] Poomsalood S., Muthumayandi K., Hambly K. (2019). Can stretch sensors measure knee range of motion in healthy adults?. Biomed. Hum. Kinet..

[30] Kramer R.K., Majidi C., Sahai R., Wood R.J. Soft curvature sensors for joint angle proprioception. Proceedings of the 2011 IEEE/RSJ International Conference on Intelligent Robots and Systems.

[31] Shen Z., Yi J., Li X., Lo M.H.P., Chen M.Z.Q., Hu Y., Wang Z. (2016). A soft stretchable bending sensor and data glove applications. Robot. Biomim..

[32] Huang B., Li M., Mei T., McCoul D., Qin S., Zhao Z., Zhao J. (2017). Wearable Stretch Sensors for Motion Measurement of the Wrist Joint Based on Dielectric Elastomers. Sensors.

[33] Al-Nasri I., Price A.D., Trejos A.L., Walton D.M. A Commercially Available Capacitive Stretch-Sensitive Sensor for Measurement of Rotational Neck Movement in Healthy People: Proof of Concept. Proceedings of the 2019 IEEE 16th International Conference on Rehabilitation Robotics (ICORR).

[34] Totaro M., Poliero T., Mondini A., Lucarotti C., Cairoli G., Ortiz J., Beccai L. (2017). Soft Smart Garments for Lower Limb Joint Position Analysis. Sensors.

[35] Kim D., Kwon J., Han S., Park Y.-L., Jo S. (2019). Deep Full-Body Motion Network for a Soft Wearable Motion Sensing Suit. IEEEASME Trans. Mechatron..

[36] Lee H., Cho J., Kim J. Printable skin adhesive stretch sensor for measuring multi-axis human joint angles. Proceedings of the 2016 IEEE International Conference on Robotics and Automation (ICRA).

[37] Mengüç Y., Park Y.-L., Pei H., Vogt D., Aubin P.M., Winchell E., Fluke L., Stirling L., Wood R.J., Walsh C.J. (2014). Wearable soft sensing suit for human gait measurement. Int. J. Robot. Res..

[38] Liao X., Liao Q., Zhang Z., Yan X., Liang Q., Wang Q., Li M., Zhang Y. (2016). A Highly Stretchable ZnO@Fiber-Based Multifunctional Nanosensor for Strain/Temperature/UV Detection. Adv. Funct. Mater..

[39] Chen S., Wei Y., Wei S., Lin Y., Liu L. (2016). Ultrasensitive Cracking-Assisted Strain Sensors Based on Silver Nanowires/Graphene Hybrid Particles. ACS Appl. Mater. Interfaces.

[40] Yi F., Lin L., Niu S., Yang P.K., Wang Z., Chen J., Zhou Y., Zi Y., Wang J., Liao Q. (2015). Stretchable-Rubber-Based Triboelectric Nanogenerator and Its Application as Self-Powered Body Motion Sensors. Adv. Funct. Mater..

[41] Liao X., Song W., Zhang X., Huang H., Wang Y., Zheng Y. (2018). Directly printed wearable electronic sensing textiles towards human–machine interfaces. J. Mater. Chem. C.

[42] Deng C., Gao P., Lan L., He P., Zhao X., Zheng W., Chen W., Zhong X., Wu Y., Liu L. (2019). Ultrasensitive and Highly Stretchable Multifunctional Strain Sensors with Timbre-Recognition Ability Based on Vertical Graphene. Adv. Funct. Mater..

[43] Liao X., Wang W., Wang L., Tang K., Zheng Y. (2019). Controllably Enhancing Stretchability of Highly Sensitive Fiber-Based Strain Sensors for Intelligent Monitoring. ACS Appl. Mater. Interfaces.

[44] Fong D.T.-P., Chan Y.-Y. (2010). The Use of Wearable Inertial Motion Sensors in Human Lower Limb Biomechanics Studies: A Systematic Review. Sensors.

[45] Cooper G., Sheret I., McMillian L., Siliverdis K., Sha N., Hodgins D., Kenney L., Howard D. (2009). Inertial sensor-based knee flexion/extension angle estimation. J. Biomech..

[46] Schwickert L., Becker C., Lindemann U., Maréchal C., Bourke A., Chiari L., Helbostad J.L., Zijlstra W., Aminian K., Todd C. (2013). Fall detection with body-worn sensors. Z. Für Gerontol. Geriatr..

[47] Lindemann U., Hock A., Stuber M., Keck W., Becker C. (2005). Evaluation of a fall detector based on accelerometers: A pilot study. Med. Biol. Eng. Comput..

[48] Winter D. (1995). Human balance and posture control during standing and walking. Gait Posture.

[49] Wu G., Xue S. (2008). Portable Preimpact Fall Detector with Inertial Sensors. IEEE Trans. Neural Syst. Rehabil. Eng..

[50] Chen J., Kwong K., Chang D., Luk J., Bajcsy R. Wearable Sensors for Reliable Fall Detection. Proceedings of the 2005 IEEE Engineering in Medicine and Biology 27th Annual Conference.

[51] Rucco R., Sorriso A., Liparoti M., Ferraioli G., Sorrentino P., Ambrosanio M., Baselice F. (2018). Type and Location of Wearable Sensors for Monitoring Falls during Static and Dynamic Tasks in Healthy Elderly: A Review. Sensors.

[52] Ma C.Z.-H., Wong D.W.-C., Lam W.K., Wan A.H.-P., Lee W.C.-C. (2016). Balance Improvement Effects of Biofeedback Systems with State-of-the-Art Wearable Sensors: A Systematic Review. Sensors.

[53] Shany T., Redmond S.J., Narayanan M.R., Lovell N.H. (2012). Sensors-Based Wearable Systems for Monitoring of Human Movement and Falls. IEEE Sens. J..

[54] Chaccour K., Darazi R., El Hassani A.H., Andrès E. (2017). From Fall Detection to Fall Prevention: A Generic Classification of Fall-Related Systems. IEEE Sens. J..

[55] Habib M.A., Mohktar M.S., Kamaruzzaman S.B., Lim K.S., Pin T.M., Ibrahim F. (2014). Smartphone-Based Solutions for Fall Detection and Prevention: Challenges and Open Issues. Sensors.

[56] Delahoz Y.S., Labrador M.A. (2014). Survey on Fall Detection and Fall Prevention Using Wearable and External Sensors. Sensors.

[57] Dai J., Bai X., Yang Z., Shen Z., Xuan D. (2010). Mobile phone-based pervasive fall detection. Pers. Ubiquitous Comput..

[58] Sixsmith A., Johnson N. (2004). A smart sensor to detect the falls of the elderly. IEEE Pervasive Comput..

[59] Mellone S., Tacconi C., Schwickert L., Klenk J., Becker C., Chiari L. (2012). Smartphone-based solutions for fall detection and prevention: The FARSEEING approach. Z. Für Gerontol. Geriatr..

[60] Adesida Y., Papi E., McGregor A.H. (2019). Exploring the Role of Wearable Technology in Sport Kinematics and Kinetics: A Systematic Review. Sensors.

[61] Hysteresis. https://www.sensorsone.com/hysteresis/.

[62] Park Y.-L., Chen B.-R., Wood R.J. (2012). Design and Fabrication of Soft Artificial Skin Using Embedded Microchannels and Liquid Conductors. IEEE Sens. J..

[63] Kim D., Kim M., Kwon J., Park Y.-L., Jo S. (2019). Semi-Supervised Gait Generation with Two Microfluidic Soft Sensors. IEEE Robot. Autom. Lett..

[64] Nyan M.N., Tay F.E.H., Murugasu E. (2008). A wearable system for pre-impact fall detection. J. Biomech..

[65] Rescio G., Leone A., Siciliano P. (2018). Supervised machine learning scheme for electromyography-based pre-fall detection system. Expert Syst. Appl..

[66] Mark C., Schall J., Sesek R.F., Cavuoto L.A. (2018). Barriers to the Adoption of Wearable Sensors in the Workplace: A Survey of Occupational Safety and Health Professionals. Hum. Factors.

[67] Jacobs J.V., Hettinger L.J., Huang Y.-H., Jeffries S., Lesch M.F., Simmons L.A., Verma S.K., Willetts J.L. (2019). Employee acceptance of wearable technology in the workplace. Appl. Ergon..

